# Association Between Specific Internet Activities and Life Satisfaction: The Mediating Effects of Loneliness and Depression

**DOI:** 10.3389/fpsyg.2018.01181

**Published:** 2018-07-11

**Authors:** Yu Tian, Shujie Zhang, Rui Wu, Peng Wang, Fengqiang Gao, Yingmin Chen

**Affiliations:** Department of Psychology, Shandong Normal University, Jinan, China

**Keywords:** specific Internet use, loneliness, depression, life satisfaction, mediating effects

## Abstract

The current study examined the associations between specific Internet activities (online shopping, pornography use, social networking site use, and Internet gaming), life satisfaction, and the mediating effects of loneliness and depression for these associations. Participants were 5,215 students (2,303 male participants, *Mage* = 16.20 years; ranging in age from 10 to 23 years) from various school types (546 elementary school students, 1710 junior high school students, 688 senior high school students, and 2271 university students) who provided self-report data on demographic variables, online shopping, pornography use, social networking site use, loneliness, depression, and life satisfaction. The results indicated that after controlling for demographic variables (gender and age) (a) loneliness and depression had fully positive mediating effects on the association between social networking site use and life satisfaction; (b) loneliness and depression played fully negative mediating effects on life satisfaction associations with online shopping, pornography use, and Internet gaming. Therefore, loneliness and depression were the underlying mechanisms that caused life satisfaction to be affected by online shopping, pornography use, social networking site use, and Internet gaming.

## Introduction

The 20th century was the century of information and communication technologies, whereas the 21st century is the Internet age in which global Internet-based information can be easily accessed (Kim and Jeong, 2017). The Internet has enhanced convenience in modern-day life and has become a crucial shopping, communication, and entertainment tool (Tsai and Lin, 2003; Arslan, 2017). However, one long-debated question is whether Internet use increases individuals’ life satisfaction, which can be defined as a global assessment of their quality of life. Some studies have suggested that the Internet enhances convenience in performing tasks, such as collecting information, making decisions, reading, writing, communicating, and sharing, and that these Internet-mediated practices may increase individuals’ life satisfaction (Reinecke and Trepte, 2014; Dienlin et al., 2017; Utz and Breuer, 2017). However, other studies have indicated that Internet use leads to pathological Internet use, which is linked to academic failure (Iyitoglu and Çeliköz, 2017), increased depression (Fayazi and Hasani, 2017; Zhao F. et al., 2017), increased loneliness (Han et al., 2017; Tian et al., 2017), increased anxiety (Fayazi and Hasani, 2017), and psychiatric disorders (Trojak et al., 2017), and these negative consequences may reduce individuals’ life satisfaction (Kwok et al., 2016; Zhi et al., 2016; Oosterveer et al., 2017).

These inconsistent conclusions on the associations between Internet use and individuals’ life satisfaction may be caused by the differences in individuals’ Internet-based activities. For example, lonely individuals can use social networking sites to establish close relationships with peer groups that may be the individuals’ primary sources of social support; support from such peer groups may reduce these individuals’ loneliness and depression and increase their life satisfaction (Reinecke and Trepte, 2014; Dienlin et al., 2017; Utz and Breuer, 2017). By contrast, numerous people use the Internet for purposes such as viewing online pornography, online shopping, and Internet gaming; these activities may exacerbate psychological and social problems, such as loneliness (Han et al., 2017; Tian et al., 2017) and depression (Fayazi and Hasani, 2017; Zhao F. et al., 2017), undermining their life satisfaction (Kwok et al., 2016; Zhi et al., 2016; Oosterveer et al., 2017). Therefore, different Internet activities may exert different potential effects on individuals’ life satisfaction. Based on the aforementioned studies, the variables of loneliness and depression were selected to investigate the various potential effects of online shopping, pornography use, social networking site use, and Internet gaming on individuals’ life satisfaction.

### Mediating Effect of Loneliness

Satici (2018) found that people with numerous Facebook friends tend to perceive less loneliness; they also have higher life satisfaction. Although this study did not consider the potential mediating effect of loneliness, it suggested that the number of strong social networking site use is negatively associated with loneliness and is positively associated with life satisfaction. Koohikamali et al. (2017) reported that school students tend to share their frustration and distress on social networking sites; they also reported that students who post on social networking sites tend to disclose more information about themselves and their emotions than they do in person. When their disclosures receive likes or comments, such as “hang in there” or “it will get better,” they feel a sense of belonging (Koohikamali et al., 2017; Ophir, 2017). This sense of belonging may make them feel that they are not isolated, which is related less loneliness (Satici, 2018); rather, that they have numerous friends. With the resulting positive life attitude, they tend to have relatively high life satisfaction (Jiang et al., 2017; Yildiz, 2017). Therefore, loneliness may have a positive mediating effect on the association between social networking site use and life satisfaction.

Studies have revealed that frequent online shopping, pornography use, and Internet gaming exert negative effects on life satisfaction. Numerous studies have found that individuals who shop online often lack basic emotional needs, especially the love and acceptance of others, and they find establishing relationships with others difficult; they often feel lonely and have low life satisfaction (Miller, 2007; Roberts et al., 2014; Bani-Rshaid and Alghraibeh, 2017). Studies have reported that habitual online pornography use can lead to negative emotions, such as loneliness (Ray et al., 2014; Butler et al., 2017); additionally, excessive pornography use also can diminish sexual satisfaction (Blais-Lecours et al., 2016; Bőthe et al., 2017; Brown et al., 2017), genital appearance satisfaction (Kvalem et al., 2014), and relationship satisfaction (Bőthe et al., 2017), which are essential components of life satisfaction. Studies have suggested that individuals who spend excessive time gaming on the Internet can become impulsive and hostile to others, and they may suffer from poor relationships with friends and family members (Bargeron and Hormes, 2017; Ren et al., 2017); therefore, they tend to perceive relatively low social support, high loneliness, and low life satisfaction. In contrast to its effect on social networking site use, loneliness tends to have a negative mediating effect on the associations of life satisfaction with online shopping, pornography use, and Internet gaming.

### Mediating Effect of Depression

Depression is a public mental health problem that occurs worldwide; it negatively influences people’s life satisfaction (Kwok et al., 2016; Zhi et al., 2016; Oosterveer et al., 2017). Scholars have long debated whether social networking site use is positively or negatively associated with depression. Frison and Eggermont (2015) found that social networking site use predicted relatively high social support, which was related to low levels of depression and high life satisfaction. However, other studies have revealed that social networking site use yielded disproportionate upward social comparison, which was associated with high depression and low life satisfaction (Chou and Edge, 2012; Kross et al., 2013; Tandoc et al., 2015). Therefore, further investigation is warranted to explain these inconsistent conclusions. Additionally, most studies have tested either the association between social networking site use and depression (Huang, 2017) or the association between social networking site use and life satisfaction (Chou and Edge, 2012; Kross et al., 2013; Tandoc et al., 2015) but have not considered that depression may have a mediating effect on the association between social networking site use and life satisfaction.

Additionally, studies have revealed the positive effects of frequent online shopping, pornography use, and Internet gaming on depression. Gallagher et al. (2017) determined that students who shop online tend to experience postpurchase guilt (shame, anxiety, and guilt) after excessive online shopping, which can increase depression. Furthermore, spending excessive time and money on online shopping may lead them to experience academic and economic pressure, which are positively associated with depression (Liu et al., 2017; Wright, 2017). Direct testing of the association between pornography use and depression has rarely been conducted; most studies have tested the association between pornography use and mental health (including sadness, suicidal ideation, suicide attempts, stress, likelihood of feeling happiness, and depression; Yu and Chao, 2016; Msc et al., 2017) or well-being (anxiety, stress, and depression; Harper and Hodgins, 2016; Mo et al., 2017), which indirectly suggests a positive association between pornography use and depression. Numerous studies have reported strong associations between Internet gaming and depression. For example, excessive time spent on Internet gaming can lead to eating disorders and sleeping disorders, which can increase individuals’ perceived depression (Canan et al., 2013; Tan et al., 2016). Furthermore, excessive Internet gaming can cause symptoms of obsessive–compulsive disorder and anxiety, which are positively associated with depression (Bargeron and Hormes, 2017; Du et al., 2017; Kim et al., 2017; Sariyska et al., 2017). Therefore, as with loneliness, we believe that depression has a negative mediating effect on the associations of life satisfaction with online shopping, pornography use, and Internet gaming.

### Current State of Internet Use in China

Internet use is among the most widespread leisure-time activities in China. China has more than 750 million Internet users, which is the world’s largest national population of Internet users; each Chinese Internet user spends an average of 26.5 h on the Internet per week. Online shopping, social networking site use, and Internet gaming were the most widespread Internet activities in China. More specifically, China had nearly 514 million online shoppers, 692 million social networking site users, and 422 million Internet gamers. Additionally, school students constituted 24.8% of Internet users; students comprise a category of Internet users in China (China Internet Network Information Center [CNNIC], 2016). These students routinely use the Internet to play computer games, communicate with others (most have Qzone and WeChat accounts) and shop (China Internet Network Information Center [CNNIC], 2016). Studies have reported that 42.7% of Internet users view pornography (Ballester-Arnal et al., 2016; Allen et al., 2017); therefore, many Chinese school students are pornography viewers.

### Present Study

The current study tested the various effects of specific forms of Internet use (online shopping, pornography use, social networking site use, and Internet gaming) on individuals’ life satisfaction and potential mechanism of the association between these specific forms of Internet use and individuals’ life satisfaction. The variables of online shopping, pornography use, social networking site use, Internet gaming, loneliness, depression, and life satisfaction were adopted, and we hypothesized that after controlling for demographic variables (age and sex), (a) loneliness had a positive mediating effect on the association between social networking site use and life satisfaction; (b) loneliness exerted negative mediating effects on the associations of life satisfaction with online shopping, pornography use, and Internet gaming; (c) depression had a mediating effect on the association between social networking site use and life satisfaction; and (d) depression exerted negative mediating effects on the associations of life satisfaction with online shopping, pornography use, and Internet gaming.

## Materials and Methods

### Participants

In total, 5500 students from five cities in China completed the self-reported questionnaires. Among these five cities, one was a first-tier city (which is divided by city’s economic level, population number and so on; first-tier city is the best city in China), two were second-tier cities, and two were third-tier cities. Two hundred and eighty-five participants were excluded from the analyses due to excessive missed responses and uniform responses, resulting in a final sample of 5215 respondents. Specifically, 546 (264 male students) students from elementary school, 1710 (822 male students) students from junior high school, 688 (303 male students) students from senior high school, and 2271 (914 male students) students from university students.

#### Design

The present study was conducted in accordance with the 1964 Helsinki declaration and its later amendments or comparable ethical standards, with the approval of the Human Research Ethics Committee of Shandong Normal University.

### Procedure

A pre-investigation from teachers, parents, and students indicated that students in elementary school under sixth grade (i) tended to not understand the self-reported questionnaire clearly, and (ii) some of them did not have WeChat and Qzone accounts; sixth grade students could understand the self-reported questionnaires clearly after instruction from their teacher or our researchers, and all of them had WeChat and Qzone accounts (the students who did not have WeChat and Qzone accounts were excluded after test).

For university students, a team of researchers from a different city came to the university to administer a series of self-reported questionnaires. Students volunteered to participate; all of them were gathered in a big classroom, and our researchers conducted a series of questionnaires a during one full class period lasting 45 min. During this period, no university-related adults were present. All of the students were required to fill in consent forms and return them. In the elementary school survey, junior and senior high school students, both headmasters of each class (the students’ headmasters could maintain class order, which could decrease the mutual influence between students) and the researchers were present. The administration of the questionnaire was conducted by a researcher during one full class period (45 min). Additionally, all of the students’ parents were notified and given the option of refusing to allow their child’s participation. Parental consent forms were distributed to all the students. Almost 99.8% of the students’ parents returned the consent forms to allow their children’s participation. At the beginning of the session, all students were informed that no one in their university would see their reports and that the researchers would not know who the students were when processing their collective reports. At the end of the session, the students were briefed about the purpose of the research and the absolute anonymity provided in the study design.

### Measures

#### Online Shopping

Online shopping was assessed using the Online Shopping Addiction scale (Zhao H. et al., 2017). Participants answered 18 items (e.g., “I frequently think about how to gain more spare time or money to spend on online shopping”; “I spend more and more time on online shopping”) on a 7-point scale ranging from 1 = “Completely disagree” to 7 = “Completely agree”. The final score was the total of the item scores, with higher scores representing higher online shopping use. The Cronbach’s α coefficient for this sample was 0.96. An confirmatory factor analysis (CFA) indicated that the standard measurement model fitted the data well: *χ^2^*(254) = 619, comparative fit index (CFI) = 0.97, normal fit index (NFI) = 0.97, non-normed fit index (NNFI) = 0.98, roots mean square error of approximation (RMSEA) = 0.06. Additionally, the McDonald’s omega = 0.81, composite reliability = 0.82 and average variance extracted = 0.86 of the scale were also calculated.

#### Online Pornography Use

The viewing of online pornography was assessed using the Online Pornography scale (Kor et al., 2014). Participants answered 12 items (e.g., “Online pornography has created significant problems in my personal relationships with other people, in social situations, at work, or in other important aspects of my life”; “I feel I cannot stop watching pornography online”) on a 7-point scale ranging from 1 = “Strongly disagree” to 7 = “Strongly agree.” The final score was the total of the item scores, with high scores representing high online pornography usage. The Cronbach’s α coefficient for this sample was 0.94. The CFA revealed that the standard measurement model fitted the data well: *χ^2^*(149) = 441, CFI = 0.97, NFI = 0.98, NNFI = 0.97, RMSEA = 0.05. Additionally, the McDonald’s omega = 0.89, composite reliability = 0.84 and average variance extracted = 0.86 of the scale were also calculated.

#### Social Networking Site Use

Social networking site usage was assessed using the Facebook Usage scale (Ellison et al., 2007). In this study, the Facebook context was changed to the contexts of Wechat and Qzone, which were most popular social networking site in China at the time of this study. Participants answered 12 items (e.g., “WeChat/Qzone has become part of my daily routine”; “I feel out of touch when I haven’t logged onto WeChat/Qzone for a while”) on a 7-point scale ranging from 1 = “Strongly disagree” to 7 = “Strongly agree.” The final score was the total of the item scores, with higher scores representing higher social networking site usage. The Cronbach’s α coefficient for this sample was 0.85. The CFA indicated that the standard measurement model fitted the data well: *χ^2^*(154) = 389, CFI = 0.97, NFI = 0.97, NNFI = 0.96, RMSEA = 0.06. Additionally, the McDonald’s omega = 0.83, composite reliability = 0.78 and average variance extracted = 0.79 of the scale were also calculated.

### Internet Gaming

Internet gaming was assessed using the Internet Gaming Disorder test (Király et al., 2015). Participants answered 10 items (e.g., “When you were not playing, how often have you fantasized about gaming, or thought of previous gaming experiences?”; “ Have you risked or lost a significant relationship because of gaming?”) on a 7-point scale ranging from 1 = “Strongly disagree” to 7 = “Strongly agree.” The final score was the total of the item scores, with higher scores representing higher Internet gaming use. The Cronbach’s α coefficient for this sample was 0.92. The CFA demonstrated that the standard measurement model fitted the data well: *χ^2^*(132) = 368, CFI = 0.97, NFI = 0.97, NNFI = 0.97, RMSEA = 0.05. Additionally, the McDonald’s omega = 0.88, composite reliability = 0.77 and average variance extracted = 0.83 of the scale were also calculated.

### Loneliness

Loneliness was assessed by the original version of the University of California, Los Angeles (UCLA) Loneliness Scale (Russell et al., 1978). Participants answered 20 items (e.g., “I always feel lonely when I am alone”; “Do you often feel that someone is willing to talk to you?”) on a 4-point scale ranging from 1 = “Never” to 4 = “Always.” The final score was the total of the item scores, with high scores representing high loneliness. The Cronbach’s α coefficient for the present sample was 0.92. CFA showed that the standard measurement model fit the data well: *χ^2^*(132) = 368, CFI = 0.97, NFI = 0.97, NNFI = 0.97, RMSEA = 0.05. Additionally, the McDonald’s omega = 0.85, composite reliability = 0.82 and average variance extracted = 0.77 of the scale were also calculated.

### Depression

Depression was assessed by the Center for Epidemiologic Studies Depression Scale (Radloff, 1977). Participants answered 20 items (e.g., “I felt that I could not shake off the blues even with help from my family or friends”; “I was bothered by things that usually don’t bother me”) on a 4-point scale ranging from 1 = “Rarely or none of the time” to 4 = “Most or all of the time.” The final score was the total of the item scores, with high scores representing severe depression. The Cronbach’s α coefficient for the present sample was 0.92. CFA showed that the standard measurement model fit the data well: *χ^2^*(132) = 368, CFI = 0.97, NFI = 0.97, NNFI = 0.97, RMSEA = 0.05. Additionally, the McDonald’s omega = 0.87, composite reliability = 0.89 and average variance extracted = 0.75 of the scale were also calculated.

### Life Satisfaction

The Chinese version of the Life Satisfaction Scale, developed by Wang and Shi (2003), was used to test students’ life satisfaction. Participants answered seven items (e.g., “How do you feel about your relationship with your friends?”; “How do you feel about your image and performance?”) on a 7-point scale ranging from 1 = “Strongly satisfied” to 7 = “Strongly unsatisfied.” The final score was the total of the item scores, with high scores representing intense life satisfaction. The Cronbach’s α coefficient for the present sample was 0.92. CFA showed that the standard measurement model fit the data well: *χ^2^*(132) = 368, CFI = 0.97, NFI = 0.97, NNFI = 0.97, RMSEA = 0.05. Additionally, the McDonald’s omega = 0.86, composite reliability = 0.84 and average variance extracted = 0.88 of the scale were also calculated.

### Statistical Analyses

SPSS 21.0 was used to conduct bivariate correlation analysis; structural equation model (SEM) analysis was conducted using MPLUS 7.0 with Robust Maximum Likelihood Estimation (Ullman, 2006). Studies have suggested that the index of *χ^2^*is sensitive to sample size, potentially leading to oversensitive model rejection (Hu and Bentler, 1999). Thus, *χ^2^* was used as the primary criterion to evaluate the model fit in the present study. In addition, RMSEA, Tucker–Lewis Index (TLI), and CFI were also used to evaluate the model. In general, RMSEA ≤ 0.05 indicates a good model fit, RMSEA ≤ 0.08 indicates a reasonable model fit, and RMSEA ≥ 0.1 indicates a poor model fit (Hu and Bentler, 1999). A TLI and CFI of >0.95 but <1, indicates a good model fit (Hu and Bentler, 1999). The chi-squared test of difference (Δ*χ^2^*) was used to compare the fit of the nested models. A non-significant Δ*χ^2^* test indicates that the two models provide an equal fit to the data, whereas a significant Δ*χ^2^* suggests that the less constrained model should be retained.

## Results

### Descriptive Statistics and Correlation Analysis

**Table 1** shows the Pearson correlations, means, and standard deviations for all the observed factors in the measurement model. Gender (1 = male; 2 = female) was positively associated with age, online shopping, and social networking site use, but was negatively associated with pornography use, Internet gaming, loneliness, and depression; age was positively associated with online shopping, pornography use, social networking site use, and loneliness, but was negatively associated with life satisfaction; online shopping, pornography use, social networking site use, and Internet gaming were positively associated each other; online shopping, pornography use, and Internet gaming were positively associated loneliness and depression, but were negatively associated with life satisfaction; social networking site use was negatively associated with loneliness and depression; loneliness and depression were positively associated with each other, but were negatively associated with life satisfaction.

**Table 1:** Means, standard deviations, and Pearson correlations of studied variables.

Variables	*M ± SD*	1	2	3	4	5	6	7	8	9
(1) Gender	1.55 ± 0.52	1								
(2) Age	16.2 ± 3.21	0.05^∗∗^	1							
(3) Online shopping	40.10 ± 20.37	0.12^∗∗^	0.25^∗∗^	1						
(4) Pornography use	19.70 ± 12.17	-0.19^∗∗^	0.08^∗∗^	0.34^∗∗^	1					
(5) Social networking site use	19.15 ± 6.00	0.09^∗∗^	0.35^∗∗^	0.37^∗∗^	0.09^∗∗^	1				
(6) Internet gaming	24.45 ± 12.93	-0.31^∗∗^	0.01	0.32^∗∗^	0.40^∗∗^	0.18^∗∗^	1			
(7) Loneliness	41.81 ± 9.09	-0.04^∗∗^	0.03^∗^	0.21^∗∗^	0.22^∗∗^	-0.07^∗∗^	0.24^∗∗^	1		
(8) Depression	17.71 ± 10.24	-0.03^∗∗^	0.02	0.32^∗∗^	0.30^∗∗^	-0.08^∗∗^	0.34^∗∗^	0.64^∗∗^	1	
(9) Life satisfaction	28.44 ± 4.89	0.02	-1.84^∗∗^	-0.15^∗∗^	-0.15^∗∗^	0.02	-0.17^∗∗^	-0.43^∗∗^	-0.41^∗∗^	1


^∗^*Coefficient is significant at the 0.05 level. ^∗∗^Coefficient is significant at the 0.01 level*.

### SEM Analysis for the Studied Variables

First, a factorial algorithm parceling strategy was used to improve the quality of the indicators and model fit. Following Rogers and Schmitt (2004), factor analysis was performed, and items of each latent variable were ranked from highest to lowest according to the factor loading size. Each parcel was sequentially assigned the remaining items with the highest and lowest rankings, with alternating directions through the parcels, until all items were assigned. For example, in the case of 20 items assigned to five parcels, Parcel #1 = items ranked 1, 10, 11, and 20; Parcel #2 = items ranked 2, 9, 12, and 19; Parcel #3 = items ranked 3, 8, 13, and 18; and Parcel #4 = items ranked 4, 7, 14, and 17; Parcel #5 = items ranked 5, 6, 15, and 16. In the present study, 20 items of both loneliness and depression were parceled in five parcels, which consisted of four items; and 18 items of online shopping was parceled in six parcels, which consisted of three items.

Second, CFA was used to test the measurement model for the latent variables (each of the latent variables was specified to covary with all other latent variables), and the parcels or items were used as the observed indicators. The model exhibited good fit indexes, χ^2^(188) = 587, *p* < 0.001, RMSEA = 0.05, TLI = 0.98, CFI = 0.98. As shown in **Table 2**, all the factor loadings of the observed indicators could significantly predict latent variables, ranging from 0.54 to 0.89.

**Table 2:** Factor loadings for online shopping, pornography use, social networking site use, Internet gaming, loneliness, depression, and life satisfaction.

Variables	Online shopping	Pornography use	Social networking site use	Internet gaming	Loneliness	Depression	Life satisfaction
Items	Factor loadings	Factor loadings	Factor loadings	Factor loadings	Factor loadings	Factor loadings	Factor loadings
1	0.74	0.88	0.56	0.85	0.82	0.72	0.65
2	0.76	0.84	0.64	0.75	0.74	0.74	0.72
3	0.81	0.79	0.54	0.69	0.75	0.69	0.74
4	0.69	0.82	0.72	0.66	0.72	0.78	0.74
5	0.74	0.89	0.74	0.74	0.67	0.77	0.66
6	0.72	0.77	0.65	0.72			0.59
7		0.82	0.72	0.76			0.68
8		0.74	0.59	0.69			
9		0.76	0.67	0.66			
10		0.77	0.69	0.78			
11		0.79	0.58	0.79			
12		0.74	0.54				


**FIGURE 1:**
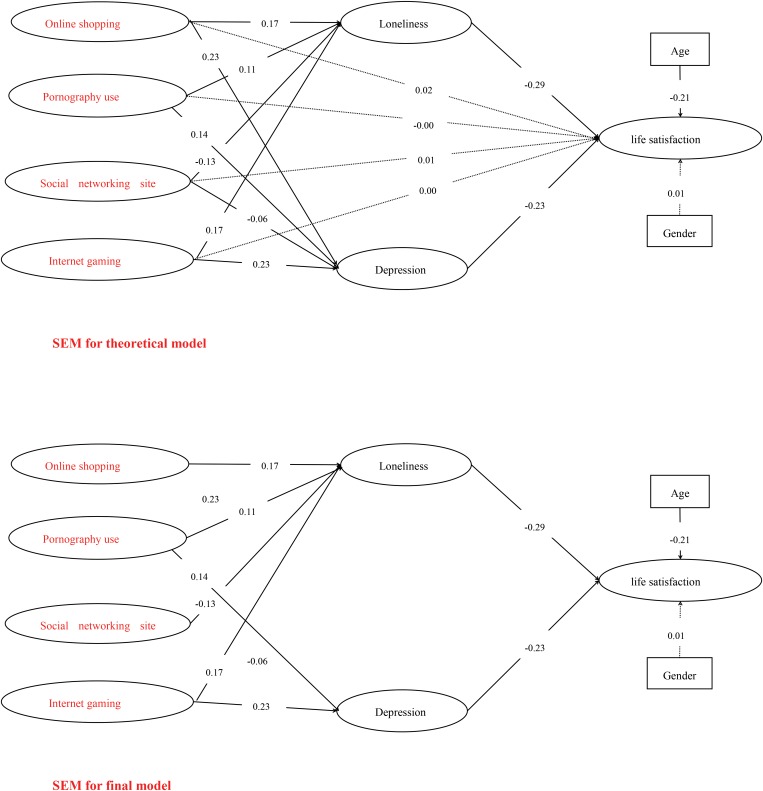
Associations among the studied variables and standardized parameter estimates of the final model. LS, life satisfaction; thick lines represent significant paths (coefficient is significant at the 0.01 level), and dashed lines represent non-significant paths (coefficient is non-significant at the 0.05 level); gender and age were controlled as independent variable.

Finally, SEM analyses was used to test the associations among online shopping, pornography use, social networking site use, Internet gaming, loneliness, depression, and life satisfaction. Firstly, we built theoretical model, which contained all the hypotheses (see **Figure 1**). All the standardized path coefficients were presented in this model [*χ^2^*(167) = 513, *p* < 0.001, RMSEA = 0.05, TLI = 0.98, CFI = 0.98]; the dotted lines were not significant, whereas the solid lines were significant. Then the final model was built that all the dotted lines were deleted [*χ^2^*(171) = 513, *p* < 0.001, RMSEA = 0.05, TLI = 0.98, CFI = 0.98]. The chi-square test of difference indicated that the final model fit the data better than the theoretical model (Δ*χ*^2^ = 4, Δ*df* = 4, *p* < 0.01). Thus, the final model was then used in subsequent analysis.

Additionally, we found that 13% of the variance of life satisfaction was explained by loneliness (*β* = -0.29, *p* < 0.01), and 12% of the variance of life satisfaction was explained by depression (*β* = -0.23, *p* < 0.01); 8% of the variance of loneliness was explained by online shopping (*β* = 0.17, *p* < 0.01), 5% of the variance of loneliness was explained by pornography use (*β* = 0.11, *p* < 0.01), 6% of the variance of loneliness was explained by social networking site use (*β* = -0.13, *p* < 0.01) and 8% of the variance of loneliness was explained by Internet gaming (*β* = 0.17, *p* < 0.01); 12% of the variance of depression was explained by online shopping (*β* = 0.23, *p* < 0.01), 7% of the variance of depression was explained by pornography use (*β* = 0.14, *p* < 0.01), 3% of the variance of depression was explained by social networking site use (*β* = -0.06, *p* < 0.01) and 12% of the variance of depression was explained by Internet gaming (*β* = 0.23, *p* < 0.01).

The aforementioned paths were indicative of some mediational mechanisms. The association between online shopping, pornography use, social networking site use, Internet gaming and life satisfaction was mediated through loneliness and depression. The significance of these mediational paths was tested using bootstrapping. With this aim, 1,000 bootstrap samples were created from the original data set by random sampling with replacement. According to the proposal of Shrout and Bolger (2002), a mediational effect is significant at the 0.05 level if the 95% confidence level does not include zero. The results indicate that all mediational paths were statistically significant: the indirect effect of online shopping on life satisfaction through loneliness(95% CI: 0.02–0.27), the indirect effect of pornography use on life satisfaction through loneliness (95% CI: 0.03–0.22), the indirect effect of social networking site use on life satisfaction through loneliness (95% CI: 0.08–0.29), and the indirect effect of Internet gaming on life satisfaction through loneliness (95% CI: 0.05–0.19); the indirect effect of online shopping on life satisfaction through depression (95% CI: 0.09–0.31), the indirect effect of pornography use on life satisfaction through depression (95% CI: 0.03–0.34), the indirect effect of social networking site use on life satisfaction through depression (95% CI: 0.04–0.17), and the indirect effect of Internet gaming on life satisfaction through depression (95% CI: 0.06–0.18). Thus, loneliness and depression played mediating effects of the associations between online shopping, pornography use, social networking site use, Internet gaming and life satisfaction.

## Discussion

In the present study, we examined the effect of online shopping, pornography use, social networking site use, and Internet gaming on school students’ life satisfaction, as well as the mediating effect of loneliness and depression underlying these associations. Our findings contribute to the literature on school students’ life satisfaction in the following respects.

First, this study found that loneliness positively fully mediated the association between social networking site use and life satisfaction. The present study extended a previous study that argued that loneliness was a potential mechanism of the association between social networking site use and life satisfaction (Reinecke and Trepte, 2014; Dienlin et al., 2017; Utz and Breuer, 2017). Additionally, Western-based studies have uncovered strong associations between social networking site use, loneliness, and life satisfaction; Facebook use (which was the most widely used social networking site in Western countries at the time) was used to represent social networking site use (Frison and Eggermont, 2015; Ophir, 2017). However, the present study used Qzone and WeChat (which were widely used in China) to represent social networking site use, and the results of the present study were consistent with Western-based studies (Frison and Eggermont, 2015). Our findings suggest that this mediational pathway might be generalizable to both Western and Eastern cultures.

Second, loneliness negatively fully mediated the associations between online shopping, pornography use, Internet gaming, and life satisfaction. This finding was notable; it may be that excessive time spent on online shopping, pornography use, and Internet gaming lead individuals to reduce their involvement in offline interpersonal interactions. A prerequisite for a state of belongingness is the existence of meaningful, mutual social relationships or ties where interpersonal interactions are relatively frequent. However, if deficiencies exist in either of these aspects, belongingness needs remain unfulfilled and people suffer loneliness (Aanes et al., 2010). Furthermore, individuals’ life satisfaction depends not only on objective metrics, such as health and economic status, but on various subjective experiences that vary by individual (Reinecke and Trepte, 2014; Dienlin et al., 2017). Loneliness is a negative emotional response to a discrepancy between the desired and achieved quality of social interactions, which can lead to low life satisfaction (Jiang et al., 2017; Unanue et al., 2017).

Third, this study found that depression positively fully mediated the association between social networking site use and life satisfaction. This result was consistent with those of some studies (Frison and Eggermont, 2015; Ophir, 2017) but was inconsistent with other studies (Chou and Edge, 2012; Kross et al., 2013; Tandoc et al., 2015). These inconsistent results may be explained by differences in sample characteristics. For example, Ophir (2017) sample had a mean age of 15.42 years, whereas the sample of Tandoc et al. (2015) comprised university students (mean age: 19.0 years). The present study revealed that an individual’s age has a negative effect on life satisfaction. It may be that the differences in the mean ages of the samples resulted in inconsistent conclusions. Furthermore, multiple operational definitions of social networking site use were applied. For example, Tandoc et al. (2015) defined Facebook use as the only type of social networking site use, but Ophir (2017) defined social networking site use multidimensionally: not only Facebook use was considered but also Twitter, LinkedIn, Pinterest, and other social networking site use, all of which were associated with school students’ daily lives. A diverse sample of social networking sites could increase school students’ life satisfaction, whereas use of a single social networking site (i.e., Facebook) could reduce school students’ life satisfaction.

Fourth, depression negatively fully mediated the associations between online shopping, pornography use, Internet gaming, and life satisfaction. This result was consistent with those of other studies (Harper and Hodgins, 2016; Bargeron and Hormes, 2017; Liu et al., 2017; Mo et al., 2017; Sariyska et al., 2017; Wright, 2017). The consensus is that excessive time or money devoted to online shopping, pornography use, or Internet gaming can cause school students to experience postpurchase guilt (such as shame, anxiety, and guilt), which is positively associated with depression (Liu et al., 2017; Wright, 2017). Furthermore, excessive online shopping, pornography use, and Internet gaming can lead students to experience academic and economic pressure, which were associated with depression (Liu et al., 2017; Wright, 2017). Similar to loneliness, depression was defined as a negative emotional response to negative life events, which can lead to diminished life satisfaction (Kwok et al., 2016; Zhi et al., 2016; Oosterveer et al., 2017).

Finally, the present study determined that age was a negative predictor of life satisfaction. This result was consistent with the results of other studies (Montepare and Lachman, 1989; Luque et al., 2017). It may be that school students tend to receive less social support as they age, which could have a positive influence on their life satisfaction (Guess and Mccane-Bowling, 2016; Chen et al., 2017). For example, students at elementary school receive substantial support from their parents and teachers because of their young age and inability (Lu et al., 2015; Guess and Mccane-Bowling, 2016; Chen et al., 2017; Gómez et al., 2017). However, self-learning is advocated in university; therefore, the interactions between university students and teachers are relatively few, and these students may receive less support from their teachers. Furthermore, numerous Chinese students leave their hometowns and move to other cities for university. The interactions between these students and their parents decrease; students may receive little support from their parents. Therefore, school students’ age was negatively associated with their life satisfaction.

### Implications for Improving Life Satisfaction

Some implications for improving life satisfaction of school students are as follows. First, parents and teachers should pay attention to the differential effects of Internet use on school students’ life satisfaction: not only the beneficial effect of social networking site use but also the adverse effects of online shopping, pornography use, and Internet gaming. Second, our findings can help practitioners understand the pathways by which Internet use affects school students’ life satisfaction to signpost possible intervention methods. For example, reducing school students’ loneliness and depression through periodic collective counseling and collective activities may be effective for increasing school students’ life satisfaction. These activities can cause students to engage in positive interactions, such as respecting, encouraging, and helping each other, thereby facilitating positive interpersonal relationships and reducing loneliness and depression. Third, parents and teachers should pay attention to relatively older students. Although these students typically function as adults, they may still require support from their parents and teachers to maximize their life satisfaction.

### Limitations and Future Directions

Our study has certain limitations of the present study must be acknowledged. First, although efforts on random selection of students from different schools were made, and a relatively large sample was recruited, our sample included only students, so our results are not generalizable to all adolescents (e.g., those who do not attend school because they are already working). Therefore, studies with representative schools and non-schools student samples are required to extend these findings. Second, the information was entirely collected by self-reported measures, so the accuracy of individual reports cannot be guaranteed, although the measures used in the present study are applied worldwide and have demonstrated adequate psychometric properties. Probably, a multi-method assessment (e.g., the reports from students’ parents and teachers) of online shopping, pornography use, social networking site use, Internet gaming and life satisfaction would have led to more valid and reliable findings. Third, the present study employed a cross-sectional design, making a causal inference challenging. Longitudinal and experimental designs enable us to make a causal inference. Therefore, experimental-design studies are warranted to validate the results of the present study. Fourthly, most of studies were aimed at test the effect of loneliness and depression on online shopping, pornography use, social networking site use and Internet gaming, however, the present study have found online shopping, pornography use, social networking site use and Internet gaming also could predict loneliness and depression, which indicated the association between online shopping, pornography use, social networking site use and Internet gaming also could predict loneliness and depression were bidirectional, and therefore, more longitudinal study designs enable us to test the bidirectional associations. Fifthly, the present study have tested the associations between excessive usage of online shopping, pornography, social networking site, Internet gaming and life satisfaction, while not the associations between moderate usage of online shopping, pornography, social networking site, Internet gaming and life satisfaction. It may be that moderate usage of online shopping, pornography, social networking site, Internet gaming may have an positive influence on individuals’ life satisfaction. For example, individuals who do not have a partner, the consumption of pornography can help sexual satisfaction, which is positive related to life satisfaction, therefore the associations between moderate usage of online shopping, pornography, social networking site, Internet gaming and life satisfaction need to be tested in the future study. Finally, pencil and paper instrument was used to measure students’ online shopping, pornography use, social networking site use and Internet gaming, which may have caused a measurement error deviation for the subsequent analysis of the mediating role for loneliness and depression because of the low precise measurements. Future study of the use of online shopping, pornography use, social networking site use and Internet gaming was suggested to do the investigation online, which was much safer and more reliable.

## Author Contributions

YT and FG wrote the manuscript and data analysis. SZ and RW conducted data analysis and interpretation of data for the manuscript. PW and YC polished the manuscript and checked the manuscript.

## Conflict of Interest Statement

The authors declare that the research was conducted in the absence of any commercial or financial relationships that could be construed as a potential conflict of interest.
